# The effect of dietary ions difference on drinking and eating patterns in dairy goats under high ambient temperature

**DOI:** 10.5713/ajas.18.0500

**Published:** 2018-10-26

**Authors:** Thiet Nguyen, Somchai Chanpongsang, Narongsak Chaiyabutr, Sumpun Thammacharoen

**Affiliations:** 1 Department of Physiology, Faculty of Veterinary Science, Chulalongkorn University, Bangkok 10330, Thailand; 2 Department of Agricultural Technology, Faculty of Rural Development, Cantho University, 3/2 street, Cantho city, 90000 Vietnam; 3 Department of Husbandry, Faculty of Veterinary Science, Chulalongkorn University, Bangkok 10330, Thailand; 4 The Academy of Science, The Royal Society of Thailand, Dusit, Bangkok 10300, Thailand

**Keywords:** Dairy Goat, Dietary Cation and Anion Difference (DCAD), Heat Stress, Meal Patterns, Water Balance

## Abstract

**Objective:**

The present study was carried out to evaluate the effect of high dietary cation and anion difference (DCAD) rations on diurnal variations in eating and meal patterns, water intake and urination patterns in dairy goats fed under high ambient temperature (HTa).

**Methods:**

Ten crossbred dairy goats during peri-parturition period were selected and divided into two groups of five animals each. Experimental diets were control DCAD (control, 22.8 mEq/100 g dry matter [DM]) and high DCAD (DCAD, 39.1 mEq/100 g DM). The composition of two diets consisted of 44% corn silage and 56% concentrate. From the 2nd week to 8th week postpartum, goats were fed *ad libitum* twice daily either with the control or DCAD total mix ration with free access to water. The spontaneous eating and drinking patterns were determined.

**Results:**

The environmental conditions in the present experiment indicated that goats were fed under HTa conditions (average peak THI = 85.2) and were in heat stress. In addition to the typical HTa induced tachypnoea in both groups, the respiratory rate in the DCAD group was significantly higher than the control group (p<0.05). Although the goats from both groups showed comparable level of eating, drinking and urination during experiment, the meal pattern and water intake were different. High DCAD apparently increased eating and meal patterns compared with the control. At week 8 postpartum, goats from high DCAD group had significant (p<0.05) bigger meal size and longer meal duration. Moreover, high DCAD appeared to increase night-time water intake (p<0.05).

**Conclusion:**

Both meal pattern and night-time drinking effects of DCAD suggested that feeding with high DCAD ration may alleviate the effect of heat stress in dairy goat fed under HTa conditions.

## INTRODUCTION

In tropical countries, dairy animals are confronted with prolonged high ambient temperature (HTa) during prolonged summers. Decreased feed intake and increased water intake (WI) during HTa have been reported in both dairy cattle and goat [1–3]. Dairy animals exposed to heat stress changed their meal patterns by increasing meal frequency, consuming smaller meals and eating for longer periods [4,5]. The reduction of feed intake during HTa may be in part due to the animal’s attempt to reduce metabolic heat production or a gut filled by water [3,6]. Supplementation with high dietary cation and anion difference (DCAD) by increasing sodium and potassium in both species increased both WI and dry matter intake (DMI) as reported by previous studies [7,8]. Further, comparable responses have also been demonstrated in dairy cattle fed under mild degree HTa [9]. An increase in DMI effect by DCAD has been described partly via the improvement of rumen function such as ruminal pH, ruminal microbial activities and fermentation [7,10].

It was well accepted that goats fed under HTa have different mechanisms and tolerance compared with cattle. Specifically, small ruminants tend to use panting rather than sweating, as in large ruminants [11]. Dairy cattle responded to acute heat stress by increasing respiration rate and body temperature [12, 13] and altering the rumen function [14]. Because goats were considered more tolerant to HTa condition compared with cattle, and because our previous results suggested that supplemented with DCAD increased water balance and influence body fluid distribution [15]. We were interested on the effect of DCAD in relation to eating and drinking behaviors and planed the present study which aimed at investigating the potential effect of higher DCAD to alleviate the HTa effect on diurnal variations in feed intake, WI, and urination patterns.

## MATERIALS AND METHODS

### Animals and management

The experiment was carried out at the Nakornpathom training farm, Nakornpathom province, Thailand. The procedures of this experiment were performed according to the guidelines for the use of animals from National Research Council of Thailand and were approved by the Animals Care and Use Committee, Faculty of Veterinary Science, Chulalongkorn University (#1531074).

Ten crossbred female Saanen goats that were in late pregnancy, and with an average body weight (BW) of 34.5±1.4 kg were selected and used in this experiment. One month before parturition, all animals were kept in individual metabolic cages in 2×1 m shaped pens with plastic floors for adaptation. During the first week after parturition, kid from each goat was allowed suckling their mother to one week after parturition (PP-1), animals were randomly divided into two groups based on BW and milk yield, with five animals in each group. They were offered two experimental diets of either control DCAD or high DCAD (control, 22.8 or DCAD, 39.1 mEq/kg dry matter [DM] respectively). The experiment was started and lasted for 7 weeks (2nd to 8th week postpartum, PP-2 to PP-8). The experimental diets contained 44% corn silage and 56% concentrate. The corn silage used in this experiment was chopped on a 5 cm. The diets were formulated as a total mixed ration (TMR) according to national research council recommendation [16]. The levels of DCAD were varied by using NaHCO_3_ and K_2_CO_3_. The ingredients and chemical compositions of the diet are presented in Table 1. Goats were milked by hand milking twice daily at 06:00 and 13:00 h. Milk yield from all goats were recorded daily throughout the experimental period. Four percent fat corrected milk (4% FCM) was calculated based on the equation of Mavrogenis and Papachristoforou [17]. TMR was offered for goat *ad libitum* (refusals always exceeded 10% of experimental diets), twice daily at 07:00 h and 14:00 h. Goats had free access to water.

### Data collection and measurements

Feed and refusal samples were collected every day throughout the experiment and divided into two parts; one half was immediately dried in the oven at 105°C until consistent to determine DM, and the remaining samples were kept frozen at −20°C until chemical analysis. At the end of the experiment, all feed samples were thawed and mixed thoroughly, and subsamples were dried at 65°C overnight (approximately 12 h) for nitrogen and ash analysis according to AOAC [18], neutral detergent fiber and acid detergent fiber using the procedure developed by Van Soest et al [19]. Sodium and potassium were measured by atomic absorption spectrophotometer (Thermo iCE 3000 series, Thermo Fisher Scientific, Beijing, China), chloride was determined by colorimetric titration and sulfate (SO_4_^2−^) was measured by spectrophotometer (UV-VIS 1800 Shimadzu, Kyoto, Japan)

The BW of the goats was measured once per week from PP-1 to PP-8. Rectal temperature (Tr), respiration rate (RR), food intake (FI), WI, urine volume (UV), ambient temperature (Ta), and relative humidity (RH) were determined once a week throughout the experimental period, and the measurements were done every 2 h during daytime. Rectal temperature was determined by a digital clinical thermometer (digital clinical thermometer C202, Terumo, Tokyo, Japan). Respiratory rate was measured by counting the movement of the flank within one minute. The measurement of FI and WI was performed by subtracting the weight of feed/water offered with the weight of feed/water refusal. The UV was collected using a plastic container and weighted at specific time points. The Ta and RH at goat barns were recorded using a thermohygrometer (Thermohygrometer, Sato, Taipei, Taiwan). The temperature and humidity index (THI) was determined according to NRC [20] as follow:

THI=(1.8×Tdb+32)-[(0.55-0.0055)×RH×(1.8×Tdb-26.8)]

where Tdb, dry bulb temperature and was expressed as °C; RH, relative humidity.

On PP-4 and PP-8 (week 3 and 7 of experimental period), eating and meal patterns were recorded continuously for 24 h using digital balance equipped to data processing software (PBA 665 & Weigh Term 231G, Mettler Toledo, Zürich, Switzerland). For each goat, the digital balance was fixed under the feed container. The balance with feed container was protected by a wooden box. The actual weight of feed containers was checked and recorded automatically once per minute using a personal computer. Meals were defined as feed removals exceeding 5 g that were separated by at least 15 minutes of non-feeding [21]. Parameters recorded were meal size, meal duration, meal frequency and inter-meal interval.

### Statistical analyses

All data were presented as the mean and standard error of measurement. Data that contain diets and time as the factors was analyzed with the repeated two-way analysis of variance. Significance of main effects was performed by the Bonferroni post test. The data of two diets were compared using an unpaired T-test. The significance was declared at p<0.05.

## RESULTS

### Environmental condition and the effects of high dietary cation and anion difference on rectal temperature, respiration rate and milk yield

The average Ta, RH, and THI at 07:00 and 13:00 h were 27.6± 0.39 and 34.7°C±0.48°C, 71.3±0.78, and 52.1±0.92%, 78.1±0.57 and 85.2±0.49, respectively. During the experimental period, there were no difference of Ta, RH, and THI at 13:00 h between the control and DCAD groups (p>0.05, Table 2). The average Tr at 13:00 h for the control and DCAD groups were not significantly different throughout the experimental period (39.6±0.13 and 39.6°C±0.10°C, p>0.05, Table 2). However, the average of RR at 13:00 h from DCAD (148±10 breaths per min) was higher than in the control (124±10 breaths per min, p< 0.05, Table 2). The average milk yield and 4% FCM from both groups were 1.51±0.14 and 1.53±0.14 kg/d. There was no significant in both milk yield and 4% FCM between groups (p> 0.05, Table 2). During the experimental period (49 days), the total milk yield and 4% FCM from control (72.06±10.92 and 71.06±8.73 kg) were not significant different from DCAD group (75.53±9.47 and 78.85±11.82 kg).

### Effects of high dietary cation and anion difference on dry matter intake and meal patterns

**Figure 1:**
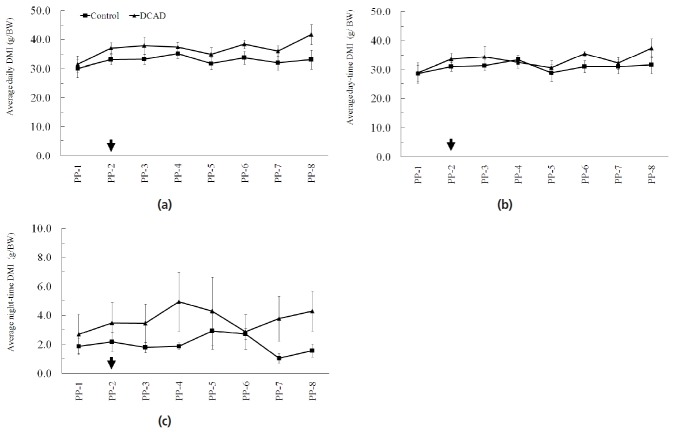
Effect of high dietary cation and anion difference on dry matter intake (DMI). Control or dietary cation anion different (DCAD) rations were provided from the 2nd week postpartum (PP-2, arrow) and DCAD effect was observed until the 8th postpartum (PP-8). The average daily DMI from both day and night time was measured manually from each week. There were no effects of DCAD on the average daily DMI (a), day-time DMI (b), and night-time DMI (c).

Under HTa condition, the average daily DMI from control and high DCAD groups were 32.7±1.8 and 36.8±1.6 g/kg BW, respectively (Figure 1a). The average DMI of day- and night-time from control and high DCAD groups were 30.8±1.9 and 33.1 ±1.8 g/kg BW (day-time, Figure 1b) and 2.0±0.4 and 3.7±1.3 g/kg BW (night-time, Figure 1c), respectively. The average total daily, day- and night-times DMI from 8 weeks during experimental period did not differ between groups (p>0.05, Figure 1a, 1b, 1c). In this current study, eating and meal pattern were also measured on PP-4 and PP-8. The 24 h DMI at PP-8 (p< 0.05), but not at PP-4 (p>0.05), from the DCAD group was significantly higher than from the control group (Table 3). This result corresponded to the larger 24 h and day-time meal sizes of DCAD group than those of the control group (p<0.05, Table 3). In addition, there were also significantly longer meal duration and less meal frequency during day time in the DCAD group than in the control group (p<0.05, Table 3).

### Effects of high dietary cation and anion difference on water intake and urinary excretion

**Figure 2:**
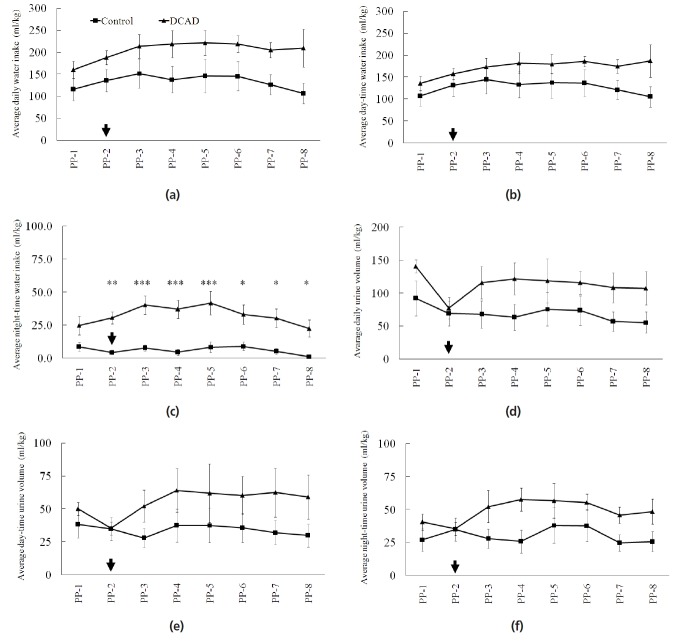
Effect of high dietary cation and anion difference on total water intake (WI) and urine volume (UV). Control or dietary cation anion different (DCAD) rations were provided from the 2nd week postpartum (PP-2, arrow) and DCAD effect was observed until the 8th postpartum (PP-8). High DCAD had no effects on the average daily WI (a) and day-time WI (b). However, the night-time WI (c) from DCAD group was higher than from the control group. There were no effects of DCAD on the average daily UV (d), day-time UV (e), and night-time UV (f). Asterisks at the specific time point indicate a significant difference within the day time. * p<0.05; ** p<0.01; *** p<0.001.

Under HTa conditions, the average daily WI from control and high DCAD groups were 133±27 and 204±18 mL/kg, respectively (Figure 2a). The average day- and night-time WI from control and high DCAD groups were 127±26 and 172±15 mL/kg (day-time, Figure 2b) and 6±2 and 32±4 mL/kg (night-time, p<0.05, Figure 2c), respectively. The averages of daily and day-time WI during the experimental period from both groups were not significantly different (p>0.05, Figure 2a, 2b). However, night-time WI was higher in the DCAD than in the control when the DCAD diet provided from weeks 2 to 8 PP (p<0.05, Figure 2c). The average daily UV from control and high DCAD groups were 69±19 and 113±19 mL/kg, respectively (Figure 2d). The average day and night times UV from control and high DCAD groups were 34±9 and 56±12 mL/kg (day-time, Figure 2e) and 30±9 and 49±6 mL/kg (night-time, Figure 2f), respectively. The average daily UV during the experimental period was not significantly different (p>0.05, Figure 2d). There was no difference in UV when day- and night-time UV were analyzed as well (p>0.05, Figure 2e, 2f).

## DISCUSSION

The data reported here revealed the effect of dietary high DCAD on eating, drinking and urination behaviors in crossbred dairy goats fed under HTa condition. Dietary high DCAD produced a positive effect on meal and eating patterns. In addition, the higher night-time WI and the non-significant difference of milk yield and urine volume apparently contributed to evaporative heat dissipation during day time.

It was clear from the environmental conditions and animal responses (Tr and RR) reported in the present experiment that all goats were fed under HTa condition and were at the stage of heat stress [6,22]. The RR reported here suggested that goats were at the early phase of panting which was thermal tachypnoea [11]. Moreover, the significantly higher RR from the DCAD over control groups in this study suggested the effect of high DCAD on acid base homeostasis. In principle, high DCAD ration via supplementation of fix cation (Na or K) with HCO_3_^−^ tends to induce alkalosis [23]. In dairy cattle, the typical acid base balance after high DCAD have been demonstrated under normal Ta and HTa conditions. Although high DCAD increased blood pH, HCO_3_^−^, PCO_2_, and RR [6–7,24,25], the indices of blood gas were within the normal range [26,27]. Unfortunately, the present experiment did not measure blood gas. It is difficult at this point to discuss the effect of DCAD on acid-base homeostasis. The significant higher RR in the DCAD over control groups suggested that under HTa high DCAD ration apparently produced an effect on the acid-base balance.

Dairy goats fed high DCAD diets tended to increase daily DMI under HTa. High DCAD ration increased week 8-PP DMI by enhancing meal size and duration. However, it is difficult at this point to make the definite conclusion that high DCAD under the HTa condition increases DMI in crossbred dairy goats as in previous reports in dairy cattle [7,24,25]. This is because there was no significant difference in daily DMI throughout the 8 weeks of the experimental period. In another point of view, the effect of high DCAD on eating and meal patterns from the present study extended the behavioral phenomenon of the DCAD effect on DMI. Similar to other mammals, ruminants use their basic taste perceptions for selecting suitable diet [28]. Behavioral responses of a tasty diet, as pre-absorptive signals, have been demonstrated in laboratory rodents as well as in humans. The analysis of eating behavior across the dietary shift revealed that rats fed a high fat and carbohydrate diet increased their eating and meal patterns within the first week [29]. An increase in meal patterns has been demonstrated within the similar period as well in crossbred dairy goat fed with fat supplemented ration [21]. Although the present results could not determine whether the pre-absorptive or the post-absorptive signals was the crucial mechanisms behind the effect of high DCAD on eating patterns [28], the effect of high DCAD on eating pattern from the current study likely came from the post-absorptive signals (satiation hormones and plasma metabolites) rather than pre-absorptive signals or palatability effect. This is because the basic taste perception effect of pre-absorptive signals could be demonstrated at the early phase of dietary shift [21,29] and when a DCAD diet was provided from the 2nd to 8th week postpartum the significant increase in meal pattern was detected at week 8-PP but not at week 4-PP.

In the current study, the patterns of WI and UV from both groups were different depending on the time of the day: day (high THI) and night (moderate THI). Animals fed high DCAD drank more water throughout the night-time, but the volume of urine from high DCAD group was not greater than the control group. Further, we also report that milk yield from both groups was not significant different. The results suggested that water retention in the DCAD group was higher than the control group during night-time. Previously, we have shown that goat fed with high DCAD had higher total body water than that in control group with no difference in extracellular fluid and plasma volume. This suggested the accumulation of water in the transcellular space of digestive tract [15]. The total water consumption values from the control and DCAD groups reported for this experiment were 4.6 and 6.8 L/d (average 5.7 L/d or 16% BW). This finding was in accordance with previous reports of goats fed under HTa conditions (9.7 L/d or 22% BW) [22] and was much greater than the level reported for goats fed under temperate zone (around 1 to 2.5 L/d, 3.0% to 5.5% BW) [30]. Further, analysis of the day- and night-time WI ratios from both groups revealed that the ratio was approximately 9:1, however, it was approximately 2:1 when goats were fed under temperate zone conditions [30]. Greater water conservation would be useful for heat dissipation mechanisms during HTa [12]. The higher total WI in response to HTa and the shift in day- and night-time WI ratio suggested that goats fed with high DCAD could improve water retention by modifying night time WI to support heat dissipation capacity.

### Conclusion

The results from current study suggested that dairy goats fed high DCAD rations used some additional behavioral strategies to ameliorate heat stress. This adjustment is considerable as an adaptive reaction to support evaporative heat dissipation under HTa conditions. Reduction of the negative effects of heat stress may in part contribute to an increase in eating and meal pattern. Taken together, the results suggested the potential positive effect of a high DCAD formulation, which may be selected as a choice of dietary strategy for ruminants fed under prolonged HTa condition.

## Figures and Tables

**Table 1:** Ingredients and chemical composition of diets

Items	Control	High DCAD
Ingredients (%)		
Corn silage	44	44
Cassava	3.26	3.26
Soybean meal	19.62	19.62
Molasses	3.69	3.69
Corn meal	25.86	24.42
Rice bran	2.25	2.25
Limestone	0.9	0.9
NaHCO_3_	0.14	0.62
K_2_CO_3_	0.28	1.24
Chemical composition (%)		
DM	35.69	35.49
CP	16.68	16.84
Ash	6.36	7.24
OM	93.64	92.76
ADF	26.12	25.16
NDF	51.61	50.03
NE_L_1) (Mcal/kg DM)	1.66	1.67
Ca1)	0.52	0.54
P1)	0.36	0.49
Na	0.10	0.16
K	1.46	1.87
Cl	0.37	0.20
S	0.13	0.15
DCAD2) (mEq/100 g DM)	22.81	39.08

DCAD, dietary cation anion different; DM, dry matter; CP, crude protein; OM, organic matter; ADF, acid detergent fiber; NDF, neutral detergent fiber; NE_L_, net energy for lactation; Ca, calcium; P, phosphorus, Na, sodium, K, potassium; Cl, chloride; S, sulphure.

1) Calculated according to NRC (1981).

2) DCAD, in miliequivalents of (Na+K) − (S+Cl)/100 g of DM.

**Table 2:** The environmental conditions and animal responses from the present experiment1)

Item		Week postpartum							

		1	2	3	4	5	6	7	8
Ta	Control2)	34.10±0.93	35.10±0.78	35.50±1.00	35.80±0.58	35.25±1.09	34.50±0.91	34.60±1.04	34.30±1.03
	DCAD2)	35.80±0.64	36.05±0.66	36.20±0.60	35.35±0.98	35.60±1.11	35.45±0.90	35.35±0.89	35.45±1.09
RH	Control2)	52.00±1.60	49.20±2.22	48.70±2.54	49.50±1.16	52.80±2.68	52.90±1.50	51.40±0.97	53.20±1.93
	DCAD2)	48.00±2.12	47.90±1.55	48.10±1.38	51.20±2.32	51.20±1.76	51.60±1.34	51.00±1.29	50.90±2.77
THI	Control2)	84.21±1.01	84.95±0.66	85.34±0.85	85.97±0.67	85.83±1.01	84.91±0.97	84.76±1.21	84.67±1.01
	DCAD2)	85.64±0.64	85.95±0.69	86.18±0.55	85.66±0.87	86.01±1.19	85.92±0.97	85.67±0.87	85.70±0.97
Tr	Control2)	39.6±0.19	39.7±0.19	39.6±0.21	39.7±0.20	39.5±0.12	39.4±0.13	39.6±0.19	39.7±0.14
	DCAD2)	39.5±0.12	39.6±0.16	39.8±0.12	39.6±0.11	39.7±0.18	39.7±0.10	39.6±0.06	39.5±0.17
RR*	Control2)	121.3±11.77	117.9±14.03	120.5±18.10	123.7±9.47	122.0±14.05	121.3±13.84	128.2±9.46	127.4±17.12
	DCAD2)	119.9±15.64	136.9±11.23	158.9±17.09	145.6±13.29	160.2±15.87	163.0±13.67	156.4±10.76	143.6±13.74
MY	Control2)	1.30±0.23	1.43±0.21	1.48±0.23	1.54±0.28	1.48±0.24	1.47±0.21	1.46±0.20	1.47±0.19
	DCAD2)	1.21±0.21	1.49±0.24	1.54±0.22	1.56±0.22	1.56±0.19	1.57±0.19	1.54±0.16	1.53±0.16
4% FCM	Control2)	1.28±0.18	1.41±0.16	1.45±0.19	1.52±0.23	1.46±0.20	1.45±0.17	1.40±0.16	1.46±0.17
	DCAD2)	1.29±0.27	1.57±0.29	1.60±0.27	1.63±0.26	1.63±0.23	1.64±0.23	1.61±0.24	1.61±0.24

Ta, ambient temperature (°C); DCAD, dietary cation anion different; RH, relative humidity (%); THI, temperature and humidity index; Tr, rectal temperature (°C); RR, respiratory rate (breaths/min); MY, average milk yield (kg/d); 4% FCM, 4% fat corrected milk (kg/d).

1) Data are presented as mean±standard error of the mean.

2) DCAD level: Control = 22.8 mEq/100 g DM; DCAD = 39.1 mEq/100 g DM.

* Significance DCAD effect (p<0.05).

**Table 3:** Effect of high DCAD on eating and meal patterns at the 4th and 8th weeks postpartum1)

Eating and meal patterns		PP-42)		PP-82)	
	
		Control3)	DCAD3)	Control3)	DCAD3)
DMI4) (g DM/kg BW)	24 hours5)	34.73±2.26	38.17±3.04	33.51±2.29	41.16±1.94*
	Day time5)	31.62±1.99	33.69±3.35	32.36±2.06	36.98±1.80
	Night time5)	1.74±0.92	4.28±1.90	1.04±0.24	4.18±1.71
Meal size (g DM/kg BW)	24 hours	2.23±0.24	2.35±0.25	2.35±0.29	3.48±0.27*
	Day time	3.05±0.48	2.89±0.40	2.93±0.26	3.98±0.14*
	Night time	0.30±0.12	0.80±0.31	0.35±0.11	1.64±0.71
Meal duration (min)	24 hours	34.60±3.61	35.40±2.29	35.00±2.70	46.40±4.48
	Day time	42.80±6.01	41.40±3.57	40.40±2.98	53.20±4.53*
	Night time	17.60±1.25	19.20±2.78	16.80±1.80	19.80±3.88
Meal frequency	24 hours	16±0.8	16±1.2	14±0.9	12±1.4
	Day time	11±1.0	12±0.7	11±0.3	9±0.4*
	Night time	5±0.7	4±0.7	3±0.7	3±0.7
Inter-meal interval (min)	24 hours	49.00±5.21	42.20±5.91	56.40±8.83	58.20±7.79
	Day time	22.40±2.50	17.40±1.29	23.20±2.48	24.60±2.91
	Night time	100.00±8.56	103.60±20.10	208.60±93.30	210.80±46.62

DCAD, dietary cation anion different; DMI, dry matter intake; DM, dry matter; BW, body weight.

1) Data are presented as mean±standard error of the mean.

2) PP-4, the 4th week postpartum; PP-8, the 8th week postpartum.

3) DCAD level: control = 22.8 mEq/100 g DM; DCAD = 39.1 mEq/100 g DM.

4) The DMI was calculated based on the sum of intake from the meal pattern measurement by automatic digital balance.

5) 24 hours from 07:00 h to 06:00 h; day time from 07:00 h to 19:00 h; night time from 19:00 h to 06:00 h.

* Significance DCAD effect (p<0.05).
